# The Story behind the Science: A history of Q-VAX, the Australian human vaccine against Q fever

**DOI:** 10.1128/mbio.00419-25

**Published:** 2025-10-30

**Authors:** Stephen R. Graves

**Affiliations:** 1 Australian Rickettsial Reference Laboratory, Geelong University Hospital, Barwon Health 3487, Geelong, Victoria, Australia; Johns Hopkins Bloomberg School of Public Health, Baltimore, Maryland, USA

**Keywords:** Q fever, vaccine, Australia, United States, history

## Abstract

Q-VAX vaccine is the only human vaccine against Q fever currently available in the world and is only available in Australia. The vaccine arose from a fruitful partnership between an American microbiologist, Richard Ormsbee, and an Australian infectious diseases doctor, Barrie Marmion. The vaccine was made in Australia from seed stock of *Coxiella burnetii*, originally isolated from an American soldier in Italy, the Henzerling strain. The vaccine was manufactured by a then Australian government-owned company, the Commonwealth Serum Laboratories, in Melbourne, using embryonated chicken eggs, starting in 1981. It was shown to be effective and relatively safe and became available for general use in 1989. It was originally used to reduce the incidence of Q fever in Australian abattoir workers but is now used (albeit underutilized) by many other occupational groups, especially in rural and regional parts of Australia. The vaccine is highly effective with over 95% efficacy, and its protective effect appears to last for many years. However, its use can lead to side effects in persons who have already been exposed to the bacterium. This has limited its use worldwide. The vaccine is currently used in Australia and, although significantly underutilized due to the need to pretest persons before vaccination, is playing a key role in reducing the incidence of Q fever in groups of Australians at risk.

## COMMENTARY

Q fever, a zoonosis caused by the bacterium *Coxiella burnetii*, is found virtually worldwide. The infection can present in several clinical forms.

- Acute Q fever is a febrile illness with severe symptoms (headache, myalgia, arthralgia, etc.) that lasts for several weeks before going into spontaneous remission and usually leading to a complete cure and immunity to reinfection.
- Chronic Q fever, which occurs months to years after acute Q fever (even after minor or asymptomatic infections), is due to reactivation of the original infection, manifesting mainly as vascular infections such as endocarditis (especially aortic valve damage) and aortitis, especially in predisposed individuals. It is difficult to diagnose and treat and has a high mortality (about 30%).
- Post-Q fever fatigue syndrome, which occurs immediately after acute Q fever, is a debilitating fatigue state that can last for months or years, with significant economic impact for patients who can no longer work.

Clearly, prevention of Q fever, a zoonosis, would be better than cure. Because the bacterium is transmitted by aerosol from infected animals (mainly goats, sheep, and cattle) to humans, classical infection control measures often don’t work well at preventing infections in the workplace. (See Box 1 for a timeline of the development of the Q fever vaccines Q-VAX.)

Box 1. **Timeline in the development of Q-VAX**

1937

Derrick recognizes Q fever as a new infectious disease in Brisbane, Australia. **Burnet**, in Melbourne, suggests the agent is a rickettsia.

1938

Davis and **Cox**, in Hamilton, Montana, isolate an infectious agent from a tick. It is virulent for guinea pigs and humans.

1940s

The Australian Q fever infection is shown to be caused by the same microbe as the U.S. tick agent, which was named ***Coxiella burnetii***.

1950s–1960s

Several experimental Q fever vaccines developed in the United States (e.g. Rocky Mountain Lab, Montana, and the United States Army Medical Research Institute of Infectious Diseases [USAMRIID], Fort Detrick, MD) and in Australia (Commonwealth Serum Laboratories [CSL], Melbourne), but none is registered for general use.

1970–1980

Barrie Marmion (in Australia) and Richard (Dick) Ormsbee (in the United States) plan to develop a Q fever vaccine for use in Australian abattoir workers. Ormsbee arranges for the Henzerling strain of *C. burnetii* to be released from USAMRIID and sent to CSL, Australia.

1981

Ormsbee visits Australia and assists CSL in manufacturing the vaccine. Marmion obtains approval to test the experimental vaccine in volunteer abattoir workers. The first batch of the new Q fever vaccine, named Q-VAX, is produced in Australia.

1982–1988

Marmion (and others) test the vaccine in five different Australian abattoirs and show it to be protective and safe in most people. Adverse reactions are seen in those persons previously exposed to *C. burneti*. Serology and skin tests used to exclude those people from vaccination.

1989

Q-VAX registered in Australia for general use.

2025

Q-VAX still in use 36 years later but only in Australia.

The infection was first recognized by Derrick in Queensland, Australia, in 1937 among abattoir workers (1). He called this previously unknown febrile illness “Q fever,” being for “query fever,” as the cause was not clear to him at that time. With the help of Burnet (2), it was shown to be a rickettsial infection. At the same time, and quite independently, in the United States, Davis and Cox (3) isolated an unknown bacterium from a tick. The bacterium was ultimately confirmed to be the cause of Q fever and subsequently named *Coxiella burnetii* (4).

This bacterium, *C. burnetii*, is highly virulent and is considered to be a select agent in both the United States and Australia because of its bioweapon potential. Hence, it must be handled in a biological safety level 3 laboratory. This was a significant practical constraint on developing a vaccine against Q fever.

## THE EARLY DEVELOPMENT OF Q-VAX

In the 1970s, Prof. Barrie Marmion, the Head of the Division of Virology, Institute of Medical and Veterinary Science (IMVS), Adelaide, South Australia, Australia, became concerned at the high incidence of acute Q fever among people working at abattoirs in South Australia. This rise in Q fever seemed to be related to the increased slaughtering of feral goats at abattoirs.

He knew that several experimental Q fever vaccines had been developed by various research groups around the world (United States, the former Soviet Union, and the former Czechoslovakia) and even in Australia at CSL, which was a manufacturer of various biologics and (at that time) owned by the Australian (Commonwealth) government. This company routinely produced antigens of *C. burnetii* (both phase 1 and phase 2) for use in the serological diagnosis of Q fever (by complement fixation test) in Australian diagnostic pathology laboratories. To protect their staff preparing these antigens from infection, CSL developed an in-house vaccine based on formaldehyde-killed Nine Mile strain phase 1 *C*. *burnetii,* and it worked. However, it was only used in-house and never commercialized.

Marmion concluded that CSL had the microbiological expertise to make a commercial vaccine for use with Australian abattoir workers, and to this end, he contacted his American colleague, Dr. Richard (Dick) Ormsbee, an expert microbiologist at the National Institute of Allergy and Infectious Diseases, Rocky Mountain Laboratory (RML), Hamilton, MT, USA, for help and advice. Ormsbee had previously prepared a Q fever vaccine (Q58A) in 1960 for in-house use at the RML, based on California milk strain 76 of *C. burnetii,* and it had been used with considerable success for many years. Those vaccinated did not get Q fever, while those who chose not to be vaccinated got Q fever at the normal background rate (5).

Ormsbee agreed to help Marmion with the development of an Australian vaccine. In a letter (24 April 1979) (6) to Marmion, Ormsbee suggested that the vaccine be made and field-tested in Australia rather than in the United States. Ormsbee had recently retired from the RML and was keen to be involved in the proposed Australian venture, while Marmion was delighted to have Ormsbee’s expertise on the project. Marmion replied (2 May 1979) (7) that he would ask CSL if it would be prepared to make the proposed vaccine and if it could then be tested in Australian abattoir workers.

Marmion discussed this proposal with Dr. Brian Feery, a research medical officer (later medical consultant) at CSL. Feery then wrote to Ormsbee (17 July 1979) (8) seeking his cooperation in the proposed vaccine project and again (29 February 1980) (9) seeking a subculture of the Henzerling strain of *C. burnetii* with which to make the proposed Australian vaccine.

The author assumes that CSL sought the Henzerling strain for this purpose, even though they already had the Nine Mile strain in-house, because the former strain had been shown to be free of adventitious biological agents. The Nine Mile strain, from which they had already prepared a vaccine for use with CSL staff at risk of Q fever, had not been so tested. Also, the U.S. experience with the Henzerling strain was more extensive, and maybe Feery felt it would be more appropriate to use the better-known strain with which to make the new Australian vaccine.

Marmion, meanwhile, obtained the agreement of CSL to make the new vaccine at their expense, and the agreement of the South Australian state government to test the new vaccine at the government-owned South Australian Meat Corporation (SAMCOR) abattoir in Adelaide. He also convinced the abattoir workers’ union to agree to their members being vaccinated, with the proviso that if anyone got sick from the vaccine, they would get 3 days off work on full pay (10)! Marmion then obtained IMVS funding for Ormsbee to travel to Australia to oversee the setting up of both the vaccine manufacturing at CSL and the field trial at SAMCOR. Ormsbee eventually arrived in Australia on 13 March 1981, for this purpose (11).

Before leaving the United States for Australia, Ormsbee made arrangements to have the Henzerling strain sent to CSL. He did this by visiting the USAMRIID at Fort Detrick, MD, where he spoke to the commanding officer (Col. Barquist) and the chief scientific advisor (Dr. Beisel) and asked them to release the strain (letter from Ormsbee to Marmion 16 May 1980 [12]). The U.S. Army had control of the Henzerling strain, as it was originally isolated from an infected American soldier in Italy during World War II. It appears that Ormsbee did not have the Henzerling strain at the RML and therefore was obliged to arrange for it to be sent to Australia from Fort Detrick. The U.S. Army had already had a vaccine made from this strain by the National Drug Company, Pennsylvania, USA, but did not use it widely. The army wanted a vaccine that could be used as a single shot, without any preliminary testing of the soldier being vaccinated (a “jab and go” vaccine).

Ormsbee wrote to Feery (16 May 1980) (13), advising him that the Henzerling strain of *C. burnetii* would be sent to him from Fort Detrick. Feery was to be sent an early egg passage 2 (EP2) preparation that had been shown to be free of all possible contaminating viruses, mycoplasmas, etc. This was the same seed that had been used to prepare the U.S. Army vaccine by the National Drug Company and had been successfully tested in army volunteers, who were mainly Seventh-Day Adventist conscientious objectors who did not want to take up arms but who were happy to have new vaccines tested on them instead.

There were a few requirements to obtaining the Henzerling strain; the Australian scientific attaché in Washington had to make a formal request to the commanding officer at Fort Detrick for the strain, following a formal request by Marmion to the attaché. A frozen preparation of the Henzerling strain would then be sent by diplomatic pouch (!) to Australia.

In his 16 May 1980 letter (13) to Feery, Ormsbee discussed his protocols for the culture of *C. burnetii* in embryonated eggs.

## THE PRODUCTION OF Q-VAX IN AUSTRALIA

The work at CSL was conducted as a national interest project by ministerial determination under S 19(1)(b)(i) with funding through S 38 of the Act, with the proviso that “should a marketable product result, the CSL Act calls for the funds obtained from the government to be repaid with interest from the profit from sales” (14).

By 17 February 1981, CSL had its first batch of eggs inoculated with the Henzerling seed *C. burnetii*. Meanwhile, Marmion was discussing some concerns about the proposed vaccine in the Federal Department of Health, Canberra (15).

After growing the bacterium, inactivating it with formalin, and partially purifying it of residual egg proteins, it was tested for sterility by egg passage.

It was agreed that the human dose of vaccine would be 30 µg, given once subcutaneously, without any adjuvant. Currently, Q-VAX contains 25 µg of vaccine per 0.5 mL dose.

Ormsbee obtained some Henzerling vaccine from USAMRIID (Fort Detrick) and sent it to Australia to be used in comparison with the new Australian vaccine, Q-VAX (11).

Ormsbee arrived in Australia in March and left in June 1981, and during this three-month period, he provided considerable assistance to Feery in making the vaccine at CSL and to Marmion in gaining approvals to carry out vaccine field tests on abattoir workers in South Australia.

The vaccine prepared at CSL, according to Ormsbee’s protocol, was used by Marmion and his co-worker to vaccinate abattoir workers in four different South Australian abattoirs and was shown to be highly protective (16–19) and in a Queensland abattoir also (20). The vaccine was considered to be no more reactogenic than any other vaccine in use at that time, provided the proposed recipient had negative serology to *C. burnetii* (phase 2 IgG was shown subsequently to be the best antibody marker of this) and a negative intradermal skin test, read at day 7, for induration (17).

In 1984, the Australian National Health and Medical Research Council Communicable Diseases Committee recommended that the new vaccine be used more widely in Australia (21). Consequently, CSL made a submission to the Therapeutics Branch of the Department of Health, Canberra, for general licensing of Q-VAX (22).

Marmion retired in May 1985 but continued to advocate for the vaccine and publish in this field, with ongoing assistance from Ormsbee in the United States.

It became clear that the vaccine required approximately 2 weeks to become fully effective. This is a typical timeframe for the human body to build up sufficient immunity to offer protection against a targeted disease after a vaccine is administered (23). If a person was infected with *C. burnetii* during the 2 week period immediately after vaccination, they may still become infected. Thereafter, however, the immunity was strong with only a few reports of vaccine failure (23, 24). Immunity was considered to be mainly due to cell-mediated immunity, as some vaccinees did not develop antibodies to *C. burnetii* following vaccination but were nevertheless immune to Q fever (16). This has an immunological basis because *C. burnetii* is an obligate intracellular pathogen, meaning that it requires a host cell to survive and to replicate within (19). An *in vitro* lymphocyte proliferation assay was more likely to be positive in vaccinees, supporting this hypothesis (17). It was more reliable than the skin test in detecting prior exposure to *C. burnetii* (by infection or vaccination) in persons but was never used routinely.

The vaccine (named “Q-VAX”) was finally registered for routine use in Australia in 1989 and has been in use in Australia ever since.

## Q-VAX, A HICCUP ON THE WAY

In 2005, CSL, by now a private company, CSL Limited, advised it had made a commercial decision to not make Q-VAX any longer.

There was great concern among rural folk in Australia about this development, as it was well recognized that Q-VAX was an important vaccine for them.

Interested professional groups (the Australasian Society for Infectious Diseases and the Australian Society for Microbiology) became involved and lobbied the ederal government, a Liberal-National party coalition, headed by Prime Minister John Howard. The author of this paper and Marmion together met the health minister, Tony Abbott, on 11 November 2005 (but not at 11 a.m.!) (Remembrance Day), in Sydney and tried to convince him of the importance of an ongoing supply of Q-VAX for the rural community.

On 18 November 2005, the author received an email from Peter Collignon, medical microbiologist at the Canberra Hospital and professor at the Australian National University (sent to all members of the Australasian Society for Infectious Diseases via their “Ozbug” IT group), in which he stated, “I rang CSL this morning, and they confirmed the decision that production (of Q-VAX) will cease and that after 2007 (when current stocks of vaccine will run out) not drinking unpasteurized milk will be an important Q fever prevention strategy.” As is well known, extremely few cases of Q fever are caused by drinking infected milk. Most cases are due to inhalation of infectious aerosols from infected animals.

Ultimately, the federal government, made up of coalition members holding rural seats in the Federal Parliament, decided that the continued availability of Q-VAX was politically important and came to an agreement with CSL to continue producing the vaccine.

On 13 April 2006, there was a media release (ABB 045/06) issued by Tony Abbott (Minister for Health and Ageing) and Peter McGauran (Minister for Agriculture, Fisheries and Forestry) stating that the government was working to secure an ongoing supply of Q-VAX. This would be done by a tender process (despite CSL Limited being the only company worldwide able to make this vaccine).

A further media release (ABB 157/06) on 30 November 2006, by the same two ministers, stated that the government had secured a continuing supply of Q-VAX. “Funding of more than $9 million will be provided to vaccine manufacturer, CSL Limited, to build a specialized manufacturing facility. CSL will manufacture the vaccine and screening tests under a 10-year contract with the Commonwealth Government.”

On 17 May 2007, the first sod was turned on CSL’s new manufacturing facility at Broadmeadows (on the outskirts of Melbourne), which would become a high-security biological safety level 3 production facility for making Q-VAX. The author attended this event with Marmion. The Cattle Council of Australia issued a media release the same day stating, “the shortage of Q-VAX over the past year or so has been of considerable concern for the industry.”

The new facility was officially opened on 1 July 2009 and named the “B.P. Marmion Q-VAX Manufacturing Facility,” with Marmion, the co-father (along with Ormsbee) of the vaccine, in attendance. The facility was opened by Richard Marles, Parliamentary Secretary for Innovation and Industry, and Mary Sontrop, General Manager, CSL Biotherapies.

Q-VAX has been periodically reviewed over the years (25–29) and always found to be highly effective at preventing Q fever, despite the problem of its reactogenicity. The latter has limited its use to Australia, as other jurisdictions have not been willing to use this vaccine, particularly during an era of vaccine skepticism.

Seqirus (previously known as CSL Biotherapies and later as CSL Seqirus but now a separate company) is expected to continue producing this important vaccine and the skin test reagent (which is simply a 1/1,000-fold dilution of the vaccine) at a new manufacturing facility built at Tullamarine, an outer suburb of Melbourne, which is due to become operational in 2026.

## CONCLUSION

Seqirus continues to be the only manufacturer of a Q fever vaccine (Q-VAX) and a Q fever skin test kit in the world to this day (2025). Generally, only people in Australia benefit from it. The rest of the world continues to suffer from Q fever without having access to a vaccine. Other potential vaccines, developed over the years, have not been registered for general use in their country of origin (30).
